# Defining the key elements of the Affolter Model® in a multiprofessional Delphi study: a first step toward evidence-based Tactual Interaction Therapy

**DOI:** 10.3389/fresc.2025.1624757

**Published:** 2025-10-10

**Authors:** Tamarith Schlunegger, Sabine Augstein, Laurent Munch, Frank Roelandt, Daniela Jakobsen

**Affiliations:** ^1^ Faculty of Psychology, University of Basel, Basel, Switzerland; ^2^ Speech Therapy and Psychology, Foundation Wahrnehmung, St. Gallen, Switzerland; ^3^ Occupational Therapy, REHAB Basel, Basel, Switzerland; ^4^ Department for Physiotherapy, VAMED Clinic Kipfenberg, Kipfenberg, Germany; ^5^ Department for Quality, Development and Education, Neurorehabilitering København, Copenhagen, Denmark

**Keywords:** Affolter Modell®, Affolter Concept®, Tactual Interaction Therapy, Delphi process, consensus, perception

## Abstract

**Background:**

Despite the versatile application of the Affolter Model®, using Tactual Interaction Therapy as a treatment approach in health, social and educational care of people with congenital brain disorder or acquired brain injury, high quality studies with robust designs for efficacy are scarce. Evaluation of the effectiveness of the Tactual Interaction Therapy requires agreement and consensus among practitioners of what constitutes this approach. Such consensus has yet to be achieved.

**Goal:**

To map the Affolter Model® by reaching consensus on its core domains among experienced practitioners.

**Methods:**

From September to December 2022, a modified online Delphi process with four survey rounds was conducted to map the core domains of the Affolter Model®. An international, interdisciplinary project group consisting of four senior instructors, trained in the use of the Affolter Model® created 29 initial statements. In the course of the four survey rounds, 40 practitioners (expert panel), all experienced users of the Affolter Model®, rated these statements on a five-point Likert scale. In addition, new statements were developed as well as new versions of existing statements. These were integrated into the surveys and submitted for evaluation. An a-priory consensus was set at a percentage approval of at least 80%.

**Results:**

Thirty-six statements out of a total of 38 statements reached consensus. The majority of statements (29/36) achieved an agreement of more than 90%. Statements that were initially rejected achieved consensus after being rephrased.

**Conclusion:**

Thirty-six core statements describing the Affolter Model® achieved consensus. By mapping the core domains of the Affolter Model®, this study provides a basis for teaching and developing the Affolter Model® in theory and practice and for further research projects to investigate its effectiveness in persons with perceptive and cognitive problems, limiting participation in everyday life.

## 1 Introduction

⁠The Affolter Model® or Affolter Concept® was developed by Affolter and Bischofberger based on the fundamental assumption that a person's development takes place in the interaction with their environment (1–4)⁠⁠⁠⁠. In this paper, the term Affolter Model is used. This model offers an approach to support children with developmental disabilities, caused by genetic diseases, unknown etiology or cerebral palsy and people with acquired brain injury and geriatric patients using physical “guidance” in the search for information to solve problems in everyday life situations⁠. In the model, this is referred to as Tactual Interaction Therapy. For all of these conditions Affolter assumes that, the underlying cause is the disorganisation of perceptual processes in the brain. This can affect problem solving in everyday life activities (ADL), learning, language and communication abilities, moving around and social interaction, resulting in limited participation and the need for help in everyday life. Members of the APW have attended courses with an examination and visit regulary meetings to improve their competencies for the work with people with perception disorders (4–7)⁠.

Depending on the situation, these are meaningful activities of daily life (ADL) from the individual's perspective. The therapeutic focus on meaningful ADLs is consistent with the ICF framework, which emphasises on participation and activity (8)⁠. Using Tactual Interaction Therapy as a treatment intervention based on the Affolter Model, the person is guided by their practitioner through the activity in a structured way. Not only with their hands, but with their entire body (4, 7)⁠. The aim is to stimulate information-seeking and problem-solving processes. The interaction with the environment involves all senses and is intended to promote the (re)organisation of brain functions and the associated acquisition or re-acquisition of skills to achieve the highest level of participation in everyday life (5, 7)⁠.

Various professional groups in health, social and education sectors use Tactual Interaction Therapy on a daily basis (6, 9, 10)⁠⁠. However, so far only few studies have assessed the effectiveness, mainly in neurological settings (11–13)⁠⁠. Studies on how changes in development and behaviour can be systematically recorded and categorised can be found in Affolter et al. (11)⁠ and Ehwald (14)⁠. The tool WESuK [Wahrnehmungsstörungen Erfassung bei Säuglingen und Kleinkindern (Perceptional Disorders Screening in Infants and Toddlers)] (15)⁠ offers one way of recording perceptual difficulties in infants and toddlers based on the Affolter Model.

Previous studies on the effect of Tactual Interaction Therapy are mainly case studies and observational studies (11–13, 16)⁠⁠⁠⁠⁠. In a single case study by Schaub et al. (13)⁠, positive effects on state of consciousness and spontaneous and goal-directed movements were observed in a patient in a minimally conscious state (MSC). Clinical behavioural changes in the form of targeted movement behaviour, reduction or cessation of hyperactivity, relevant adjustment of tone and adjustment of gaze direction (in relation to the performed activity) were observed using Tactual Interaction Therapy in the observational study by Blak Lund et al. (12)⁠⁠ with five patients with acquired brain injury. The results of these papers indicate that Tactual Interaction Therapy is associated with positive effects on patients' behaviour (11–13, 16)⁠⁠⁠⁠⁠. Lipp et al. (17)⁠ apply short- and long-term observations of neurological patients after acquired brain injury assessing patients' memory abilities, social behaviour, performance of ADL. A randomized controlled study by Latham and Stockman (16)⁠ with 34 children between the ages of 4 and 14 with autism spectrum disorders investigated the effect of Tactual Interaction Therapy on learning new words related to an ADL (pressing orange juice) and the execution of the ADL. Results showed that the application of therapy according to the Affolter Model (tactual condition: children were guided to execute the activity and received tactile support for word production) lead to a better output in the selected performance areas compared to the control group, where the children watched another person perform the ADL and heard the corresponding words. Latham and Stockman concluded that the learning effect is greater with tactile support (using the Affolter Model) than with the conventional, visual-auditory approach.

All published research to date tends to show positive effects of Tactual Interaction Therapy (12, 13, 16, 17)⁠⁠. However, these studies all have small samples, which is why Schaub et al. (13)⁠⁠ and Blak Lund et al. (12)⁠⁠ call for further studies with larger samples of different patient groups.⁠

The current lack of evidence of effectiveness is problematic in view of the increasing demand in the health and social care sector for scientific proof of effectiveness and quality of interventions. The WHO published a Guide for Evidence based decision making in 2022, pointing out the pivotal role of evidence for effective health service and reasonable use of resources and capacities in improving the effectiveness, efficiency and equity of health policies and interventions (18)⁠.

According to the definition of the Medical Research Council, Tactual Interaction Therapy can be defined as a complex intervention as it contains several interacting components, an innate flexibility and requires expertise. Recently, an extensive update and advancement of the framework was published (2021), which restated the definition of a complex intervention, to be related to the properties of the intervention, such as the number of involved components, the settings or levels targeted, range of behaviours targeted, the level of flexibility of and in the intervention or components, and the expertise and skills required (19)⁠⁠.

Practitioners must employ a high degree of flexibility and variation to treat people with different symptoms, severity of impairment and context. The desired outcome may also vary between individuals. Evidence on effect of complex interventions must indicate whether they are effective in practice and determine the active components leading to the effect. This challenges conventional randomised trials and requires alternatives that comply with the requirements of robust and valid research designs with minimal risk of bias. In order to conduct research on the effect of complex interventions, the underlying theories and content of those interventions must first be defined (19–21)⁠⁠.

For future studies on the effectiveness of Tactual Interaction Therapy, it is essential to establish consensus among practitioners regarding the content and core domains of the Affolter Model as a first step. In addition, such a description of the Affolter Model can form a strong basis for teaching of interprofessional colleagues at courses and for further education and training. Utilising a consensus process aims to clarify if the content can be retained and taught and what aspects need to be discussed, maybe adopted and developed further. The aim of this Delphi study was to reach consensus among the expert panel by mapping and describing the Affolter Model and the therein integrated principles, methods and techniques.

## 2 Material and method

### 2.1 Design

This investigation was designed as a modified Delphi study. This included establishing an interdisciplinary project group in the Affolter Model from three different countries (Germany, Switzerland (German and French speaking parts) and Denmark) with four rounds of online questionnaires. The Delphi method is an established procedure in the social and healthcare sectors for developing guidelines and integrating expert knowledge (22, 23)⁠⁠⁠. Statements pre-written by the project group were used in round one, with the possibility of revision and rephrasing by the expert panel.

### 2.2 Participation

#### 2.2.1 Recruitment

Specific members of the Arbeitsgemeinschaft pro Wahrnehmung (APW) have been defined as the expert group for this study (cf. Table 1). The APW is an association of professionals such as teachers, occupational therapists, speech and language therapists, relatives and interested members who work with and/or teach the Affolter Model and who are committed to finding solutions to problems associated with perceptual disorders. The APW is based in Switzerland and has European-wide members from Switzerland, Germany, Austria, Denmark, France, and Italy.

**Table 1:** Demographic variables and characteristics of study participants.

Demographics	Mean (sd)	Range
Age	52.9 (11.14)	31–73
	n	%
Total	40	100
Gender		
Female	31	77.5
Male	9	22.5
Nationality		
Switzerland	21	52.5
Germany	17	42.5
Other	2	5
Language of participation		
German	36	90
French	4	10
Occupation		
Occupational therapy	15	37.5
Speech therapy	6	15
Physical therapy	5	12.5
Curative Education	5	12.5
Other (psychology, special education, social pedagogy, pedagogy, supervision and management)	9	22.5
Qualification in the Affolter Model		
Therapist	19	47.5
Referent	6	15
Senior- Instructor	13	32.5
Unknown	2	5
Level of employment		
100%	14	35
80%–100%	3	7.5
60%–80%	13	32.5
>60%	6	15
Retired	4	10
Years of experience in the application of the Affolter Model		
5–10 y	9	22.5
10–15 y	3	7.5
15–20 y	4	10
20 or more	24	60

#### 2.2.2 Ethical considerations

All potential participants received written information about the study. Informed written consent was obtained prior to the start of the survey. Upon consent, each participant was assigned a five-digit numerical code.

#### 2.2.3 Inclusion and exclusion criteria

Participants eligible for the study's expert panel were required to be a member of the APW with completion of a six-week basic course in the Affolter Model including subsequent certification of qualification as a therapist in the Affolter Model, or a valid licence as an instructor or senior instructor in the Affolter Model. Furthermore, practical application of Tactual Interaction Therapy for at least five years was required. Exclusion criteria were invalid instructor or therapist license. There was no age limit.

Participants who did not take part in a survey twice, despite having agreed to participate, were not contacted further.

### 2.3 Procedure of the Delphi study

#### 2.3.1 Development of the statements

**Figure 1:**
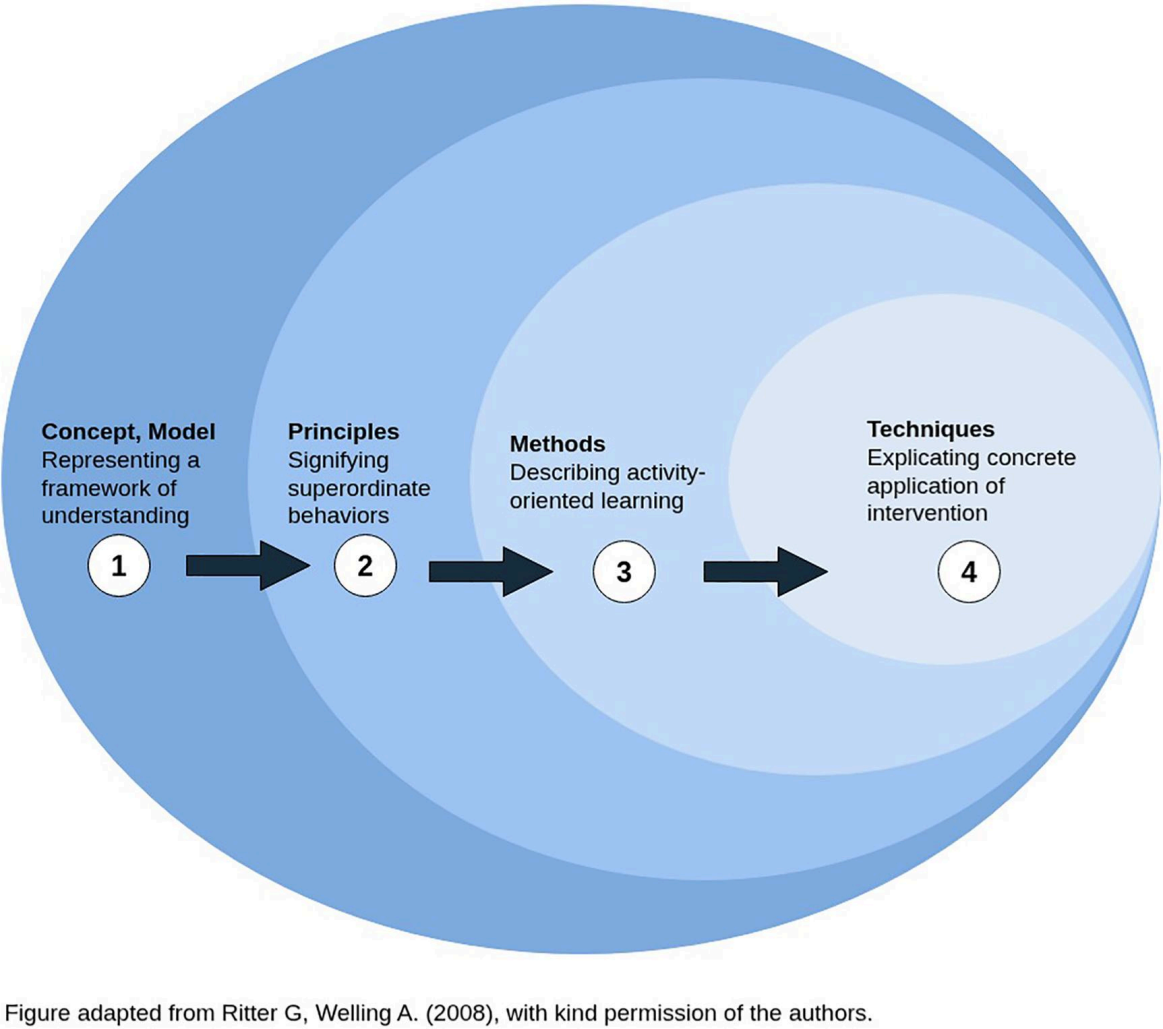
Organisational system with the core domains.

An interdisciplinary project group, consisting of four certified senior instructors from Switzerland, Germany, Denmark, and France, constructed 29 statements in advance of round one of the surveys for the expert panel. These were revised by the project group in between each new survey round, and the wording was adapted until agreement was reached within the expert panel. Specialist literature on the Affolter Model and the project group's own expertise were used to create the statements. The statements should map the basic conceptual ideas and assumptions of Tactual Interaction Therapy and the principles, methods and techniques contained therein (cf. Figure 1). In the subsequent Delphi process, which aimed to achieve consensus on the statements, these initial statements were assessed and modified by the expert panel. In each new round of the survey, the statements were organised according to the classification system by Ritter and Welling to provide a clear overview for the participants (24)⁠.

The *Concept* level comprises model-specific terms, basic statements and principles as a framework for understanding. The concept shows the connections to related sciences. The *Principles* level is characterised by the questions of which value orientations, convictions, design ideas and superordinate courses of action determine therapeutic practice. The statements assigned to the *Methods* level are subject to the question of which behaviour structure the therapeutic processes and which therapeutic actions are used to achieve the goals. The fourth level of *Techniques* comprises specific “tools” of application and are embedded in a methodical-structured approach (24, 25).⁠

#### 2.3.2 Realisation

**Figure 2:**
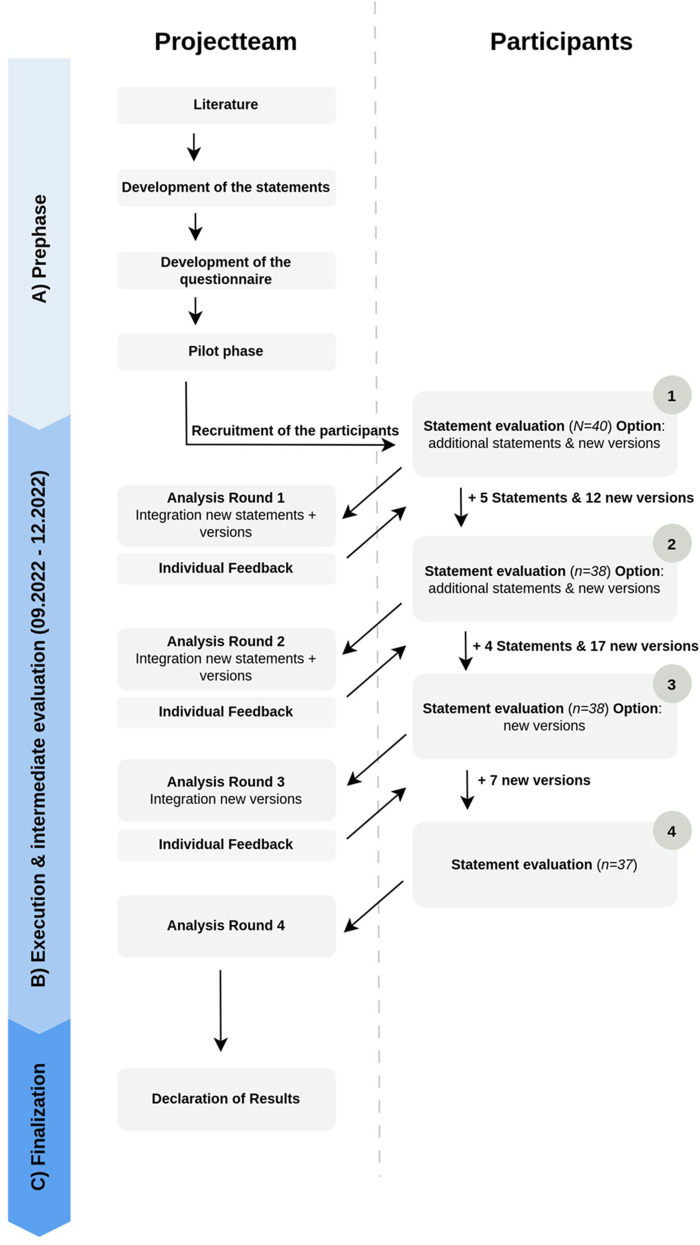
Procedure of the examination.

The expert panel accessed the survey online using the software LimeSurvey (26)⁠⁠. A deadline of one week was provided for completing one round of the survey. Members were instructed to answer the survey by themselves and not together with colleagues. The first survey was conducted in September 2022. Subsequently, the following surveys took place at one-month intervals (see Figure 2).

The expert panel was offered to take part in all four surveys in German or French. The translation was provided by two native speakers. In the first round of the survey, participants were introduced to Ritter and Welling's (24)⁠⁠ categorisation system with the help of illustrations and explanatory text (cf. Figure 1). The statements were presented in the survey according to the level to which they belonged. Agreement or disagreement with a statement could be indicated on a five-point Likert scale (0 = strongly disagree, 1 = somewhat agree, 2 = moderately agree, 3 = fairly agree, 4 = strongly agree). In addition to evaluating the statements, participants had the opportunity to create new statements. A text field at the end of the survey allowed participants to comment on the survey or on individual statements. Demographic information on participants was requested in the first survey.

Between the first and second round of the survey, the results were analysed and reported back to the expert panel by individual feedback (see section Feedback). Statements with an agreement rate of over 80% were accepted (see section Data Analysis). Accepted statements were not included in the subsequent rounds. Statements with less than 80% agreement were classified as rejected and resubmitted for voting in the follow-up round. New statements, proposed by the expert panel, were included in the second round of the survey. Due to the numerous suggestions for changes to the statements made by the expert panel, the decision was made to accept suggestions for rephrasing existing statements into new versions.

In the summary of the results of the second round of the survey, participants were again able to vote on a five-point Likert scale regarding newly created statements as well as those rejected in the first round. In cases where several similar versions of a statement existed, participants could choose their preferred version. As before, it was also possible to propose and submit suggestions for new statements or revised versions.

From the third survey round, it was only possible to create new versions of existing statements (cf. Figure 2). Similar to the phase between the first and second survey, the percentage agreement of the respective statements was calculated between the third and fourth round and again reported back to the participants individually.

#### 2.3.3 Feedback

After completing each round of the survey, all participants received individualised feedback on the results, presented as an Excel spreadsheet (see section Procedure of the Delphi study). This overview illustrated the level of agreement for the respective statements. The level of agreement was given as a mean percentage value. In addition to the mean percentage value, the individual voting behaviour was also shown. This allowed the participants to anonymously compare their answers with those of the other participants, reflect on them and adjust their answers in the following round (27)⁠.

### 2.4 Data analysis

Mean values were converted into percentage values to determine agreement. The value 4 corresponds to full agreement, which means 100%. A Statement was considered accepted if the weighted average was at least 3.2, which means at least 80%. In addition to the mean value, the median value was also taken into account.

When several versions of a statement were suggested, the participants were asked to decide in favour of one version of a statement. In some cases, new versions of rejected statements (*M* < 80%, *Mdn* < 4) were created within the same round. When this occurred, the new versions of the rejected statement were presented for voting in the subsequent round. It was also possible to reject all versions of the statement.

### 2.5 Anonymisation of the data

In order to ensure the anonymity of the participants, each person was given a five-digit numerical code for the entire survey. This code was known only by the study manager and was used to ensure that individual feedback could be provided after each round of the survey.

## 3 Results

### 3.1 Participants

Seventy-one potential participants were eligible and contacted by APW and asked to be part of an expert panel in this study. Subsequently, 40 participants answered the first survey (56.5%) and started the Delphi process. In the second and third round, the participation rate was 38 people (95%). In the fourth and final round, 37 people (92.5%) were involved. The demographic details of the participants are shown in Table 1. The majority of participants were female (*n* = 31) and employed in Switzerland and Germany. The sample was made up of a heterogeneous professional group from the health, social and education sectors. The average age was 52.9 years.

### 3.2 Development of the statements and revisions

The first round of the survey contained the 29 initial statements developed by the project group. Over the four survey rounds, the expert panel created a total of 9 new statements and 36 new versions of 15 accepted statements (cf. Figure 2). A total of 38 statements and 36 different versions were therefore assessed. Of the 38 statements, twelve statements each represent the Concept level (cf. Table 2) and Principles (cf. Table 3). The Method level contains five statements (cf. Table 4) and the Techniques level contains nine statements (cf. Table 5).

**Table 2:** Organisational level concept*.*

Code	Statements from the organizational level concept	Status
K1	Insight into normal child development, with regard to tactual interaction in everyday life, perceptual organisation, movement, language, communication and social interaction, is an important base of the Affolter Model®.	Accepted K1
K2	Tactual interaction experiences in everyday life events represent the root of human development (root model).	Accepted K2
K3	A repertoire of tactual interaction experiences in everyday life leads to the growth of the root as a prerequisite for the development of the so-called branches within the root model, such as e.g.: social skills.	Accepted K3
K4	There are connections between interaction, information and the associated organisation of perception. These are essential bases *n* of the Affolter Model®.	Accepted K4
K5	*Knowledge from the psychology of perception, in particular about the role of tactual interaction experience within the intermodal organisation of perception, is an indispensable foundation of the Affolter Model®.* • Version K5a	Accepted K5a
K6	*We endeavor to integrate theories from various related sciences and relevant findings (neurorehabilitation, Internal Classification of Function (ICF), embodiment, neuroplasticity, speech development psychology, learning psychology) into the further development of the Affolter Model®.* • Version K6a	Accepted K6a
K7	Within the Affolter Model, the tactile-kinesthetic system considered particularly important in the organisation of perception. It is the only sensory system that humans use simultaneously to search for information and to interact within the environment.	Accepted K7
K8	*The situation (location with environment and people and the design of the current place of activity) in which a person finds themselves has a significant influence on the organisation of information (perceptual performance).*	Accepted K8
K9	*Tactual interaction is important for the organisation of perception. And improved or developed perceptual organisation is equating synonymous with learning. Learning, in turn, means working in everyday life—for a self-determined life. The development of independence, along with personal, social and methodological skill fosters to educational competencies and a self-determined life.* • Version K9a; K9b; K9c	Rejected
K10	*The tactual interaction between a person and it´s environment is the base of the Affolter Model.* • Version K10a; K10b	Accepted K10a
K11	*Learning in everyday life is a defining feature of the Affolter Model because it plays a decisive role for the brains networking and is therefore very different from training of single functions.* • Version K11a; K11b	Accepted K11a
K12	*Tactual Interaction Therapy is used for the assessment and treatment of adults and/or children with developmental and/or behavioural disorders, acquired or progressive brain damage, with limitations in language and communication, in the planning of movements and daily life activities and their execution.* • Version 12a	Accepted 12a

The statements shown in italics are statements created by the participants.

**Table 3:** Organisational level principles.

Code	Statements of the organizational level Principles	Status
P1	*To assess the developmental status of an individual, especially in terms of their production abilities, observations are made in various situations, including spontaneous behavior and problem-solving during daily activities. Additionally, to evaluate a person's understanding, observations during guided interactions are also part of the assessment.* • Version P1a; P1b	Accepted P1b
P2	Behavioral observations (see Statement 1) are interpreted to understand the organization of perception and the individual's interaction with their environment. Additionally, these observations help assess the person's level of understanding and problem-solving ability in activities of daily living (ADL).	Accepted P2
P3	The evaluation of the (developmental) status forms the basis for selecting and organizing the situation. This includes choosing an appropriate activity of daily living (ADL), determining the type of/ kind of guiding, arranging the environment, and selecting the suitable/ appropriate positions for the affected person.	Accepted P3
P4	Clinical reasoning: The analysis of behaviour in various situations leads to a conclusion regarding the main problems of the affected person. This is a prerequisite for formulating therapy goals and determining the specific course of intervention The achievement of these goals is continuously evaluated and next steps are adapted*1.	Accepted P4
	*1: Clinical reasoning refers to thinking and decision-making processes within a therapeutic setting.	
P5	The therapy is designed to stimulate information-seeking and problem-solving processes.	Accepted P5
P6	Affected individuals are engaged at the level of understanding, where developmental and learning processes initially begin. Understanding is considered more comprehensive than the production stage. On the path from understanding to production, two additional stages exist: recognition and expectation.	Accepted P6
P7	*Video recordings of the assessment/diagnostics and therapy sessions are made to analyse the recorded observations in more detail. Of primary interest are observations of behaviour, which are used and interpreted with regard to the organisation of perception and the complexity of the interaction. Equally important are persistent changes in behaviour, which are used to understand the respective structure of the ADL.* • Version P7a	Accepted P7a
P8	*Observations from video recordings of findings/diagnostics and therapy sessions in different situations can be presented and summarised in the form of scripts, windows, flow charts and structural images. Due to time constraints. The detailed analysis described above is only possible to a limited extent in the daily routine of rehabilitation and other therapeutic settings.* • Version P8a, P8b	Accepted P8b
P9	Relevant and meaningful ADL are organised with the affected person. The familiarity of the activity and/or situation is adapted and varied depending on the developmental stage of the affected person. • Version P9a; P9b; 9c	Accepted P9
P10	In the Affolter Model®, an interprofessional team ideally surrounds the affected individual. Each member contributes their specific core competencies, working collaboratively while learning from, with, and about one another.	Accepted P10
P11	In the Affolter Model, relatives/carers/family members are involved and instructed as far as possible and relevant.	Accepted P11
P12	*Working with familiar and unfamiliar events is often a balancing act. When guiding affected people, it is important to always be aware of what is known and what is unknown in order to anticipate what might be complicated for the person concerned. The occurrence of stress is an indicator of how well the person understands and can be overcome difficulties within the activity. If necessary, for example, the structure of the interaction can be simplified.* • Version: P12a; P12b	Rejected

The statements shown in italics are statements created by the participants.

**Table 4:** Organisation level methods.

Code	Statements of the organizational level Methods	Status
M1	*ADL are carried out with the affected person in a non-verbal way to provide tactual information about the ADL and the position. Any problems that arise are solved with the affected person wherever possible. This is done at the level of understanding.* • Version M1a	Accepted M1a
M2	*The diversity and varying complexity of ADL are used to expand the repertoire of the affectet person when solving problems and to stimulate information-seeking and problem-solving processes as well and (facilitate the formation of hypotheses) the establishing of hypotheses* • Version M2a	Accepted M2a
M3	*ADL or parts of them are verbalized after guiding. The affected person is offered different forms of the experienced content. This allows a person to get access to the stored experiences (content) of tactual interaction and link these to the forms offered.* • Version M3a	Accepted M3a
M4	Non-verbal problem solving in an ADL can take place individually or in group situations, depending on the current status/needs of the affected person.	Accepted M4
M5	*Relatives can be involved by participating in therapy, self-experiences and practical instruction. Support for relatives can include guiding, ideas for modifying/adapting the environment, starting an ADL in a certain situation as well as interpreting the affected persons behaviour together.* • Version M5a	Accepted M5a

**Table 5:** Organisation level technique*s.*

Code	Statements of the organizational level Techniques	Status
T1	In therapy (and also in everyday life), the affected person is offered an environment that is as natural and stable as possible. This can also mean that the environment is specially *arranged*, depending on the current condition of the affected person (e.g., creating niches). Depending on the situation, helping aids are used (e.g., stable positioning material).	Accepted T1
T2	The treatment offered takes place in real life context. The selected ADL are meaningful for the affected person and appropriate to their individual everyday life and needs.	Accepted T2
T3	*When considering the selection of suitable ADL, the aspect of the so-called perceptibility of changes of topological relationships plays an important role. This aspect is also considered when selecting the necessary objects.* • Version T3a	Accepted T3a
T4	*Different types of guiding are used (nursing guiding, elementary guiding, intensive guiding). The choice for the type of guiding depends on the status of the person affected and the selected ADL. Intensive guiding is used almost exclusively in therapeutic settings.* • Version T4a, T4b	Accepted T4b
T5	Different forms and types of presentation are used when verbalizing/providing language, depending on the level of understanding of the affected person.	Accepted T5
T6	*The technique of guiding includes: the use of the stable environment (niche), selection of the appropriate beginning of the activity, finger-hand coverage (if necessary finger-finger- coverage in elementary guiding), systematic change of searching for information of the “WHAT” (ADL) and “WHERE” (position), attention to the “train-station phenomenon”, systematic change of sides between the right and left side of the body, stuffing in nursing guiding, inclusion of meaningful and targeted position variations or changes of the position within the ADL.* • Version T6a	Accepted T6a
T7	*So-called “magic” (execution of parts of the ADL by the caregiver) is avoided, i.e., all topological changes are offered tactually to the affected person during the guided ADL. Magic may and should be used before the guided activity, e.g., objects are brought to the table before the start.* • Version T7a; T7b; T7c	Accepted T7a
T8	*If the quality of production is sufficient, the affected person may be able to perform partial steps of the ADL independently. It is important that the therapist immediately starts to guide again, if the quality declines (e.g., due to perseveration, hectic movements, increased tension) or if the affected person has no hypothesis for the next step in the ADL.* • Version T8a	Accepted T8a
T9	*In elementary guiding, it is important to involve not just the arms and hands but also the legs and feet, when relevant to the ADL, to make topological changes perceptible or to provide stability to the affected person* • Version: T9a; 9b	Accepted T9b

All final statements from the survey are listed in the tables below (Tables 2–5). The statements shown are the final statements, including new versions of original statements. Statements for which there was no consensus are also shown in the respective levels.

### 3.3 Analysing consent

When analysing agreement, both the mean percentage agreement and the median value were considered. Twenty-nine of the 38 statements in total reached an agreement rate of at least 90%. This means that agreement with these statements was above the defined threshold of 80% (*Mdn* *=* 4). Furthermore, three statements achieved sufficient agreement with an approval rate of over 80% and a median of four. For 15 statements of these 32 accepted statements, new versions were also developed by the participants and submitted for voting. For all 15 statements, a new modified version received the highest level of approval compared to the original version. A detailed overview of the specific level of approval for the individual statements can be found in Supplementary Appendix S1.

The six statements: K6, K9 (Table 2), P8, P12 (Table 3) and T7 and T8 (Table 5) were rejected by the expert panel during the first presentation and the first follow-up round. Only the modified version of these rejected statements led to acceptance of four of the six statements. For statements K9 and P12, the approval rate for all versions was below 50% (see Supplementary Appendix S1). The lack of a clear majority was categorised as a rejection.

## 4 Discussion

### 4.1 Summary of results

The Affolter Model is a developmental model with a therapeutic approach derived from it (Tactual Interaction Therapy). Affolter considers tactual interaction experience between humans and their environment when solving problems in everyday life as the root of human development (6)⁠. The results of Affolters various cross-sectional and longitudinal studies led to the formulation of this developmental model (1, 28)⁠.

Previous case studies show that treatment using Tactual Interaction Therapy is associated with positive effects on the state of consciousness, recovery of abilities ⁠⁠ (13, 17)⁠ and positive clinical behavioural changes⁠⁠ (12, 16, 17)⁠. A standardised description of the content of the Affolter Model is necessary for future studies on effectiveness to meet the requirements for quality of evidence. In the present study, the core domains: concept, principles, methods and techniques of the Affolter Model were identified for the first time in using a Delphi process.

Participation in the four survey rounds was high and always exceeded 90%. The significantly higher proportion of female participants in this survey is representative of the greater professional representation of women in the health and social care sector (29, 30).

Of the total 38 statements, 32 statements achieved agreement above the defined threshold of 80%. The high level of agreement in this study is comparable with the results of other Delphi studies in neurorehabilitation (31, 32)⁠⁠⁠. The fact that the statements had already been developed and revised in an exchange between four senior instructors may have favoured the high level of agreement within this study. In the case of the statements with agreement below the threshold value, the majority of new versions received sufficient agreement. This is presumably due to the fact that the new versions expressed the content better than the original versions through rewording and additions. Only statements K9 and P12 and their new versions did not reach agreement. Possible reasons for this could be that *stress* and *learning* are terms that are difficult to define. In addition, several new versions of both statements were available for assessment (Supplementary Appendix S1), which could have made it more difficult to reach a consensus.

### 4.2 The core domains of the Affolter Model

The following section presents the statements that proved to be representative of the Affolter Model in this study. Firstly, the statements are listed.

#### 4.2.1 Concept

The concept level contains statements on model-specific terms, basic statements, and principles (statements in Table 2). There is agreement among the participants that the basis of the Affolter Model is the tactual interaction between the person and the environment (K10). Tactual interaction is seen as the root of human development (K2); (4, 6, 7, 11)⁠⁠⁠⁠. A repertoire of tactual interaction experiences is the prerequisite for further development (K3); (4, 7)⁠. Statements K2 and K3 refer to the underlying development model. In this model, Affolter (7)⁠⁠ makes an analogy with a tree: »When a plant has *sick roots*, it will not become healthy when we treat the leaves or the branches. (…) We must work at the root. We must treat the cause and not the symptoms« (p. 165) (Table 2).

The Affolter Model emphasises learning in everyday life and thus distinguishes it from task-specific training, such as repeatedly practising making coffee (K11) (4)⁠⁠. Regarding practising individual functions in statement K11, Affolter postulated: »When a *situation changes, the performance deteriorates rapidly.* (…) Then we hear complaints about poor transfer of the performance.« (7)⁠, p. 138. This is why everyday life, as described by Ott-Schindele (9)⁠⁠, is ascribed particular importance: »Everyday life with all its complex demands offers the ideal framework for therapeutic intervention—but of course also for consolidating experiences in constantly changing situations and gaining confidence in execution«, p. 421 [Originally written in German, translated into English by the author]. With the help of statement K9, a more detailed attempt is made to define the concept of learning in the Affolter Model. Statement K9 was rejected and none of the versions reformulated by the participants achieved consensus. The disagreement among the participants indicate that the content of this statement is complex and should be revisited in more detail in the future.

Knowledge of child development, social interaction, movement, language, communication (K1) and the psychology of perception (K5) is considered an important basis for the Affolter Model (33)⁠⁠. As early as the 1970s, Affolter and colleagues demonstrated the importance of sensitivity in child development by observing the behaviour of children with speech difficulties (6)⁠⁠. It is therefore not surprising that development, communication and language are recognised as important foundations of the Affolter Model in this study. With regard to statement K5, more detailed explanations can be found in Sergi (33)⁠⁠, who looks at sensibility as a bridge between perception, action and cognition.

There is a consensus in this study that the organisation of perceptual performance is influenced by the situation in which the person concerned finds him/herself (K8), the interaction and the information available (K4). This is illustrated by an everyday example: I walk down the street and look in the shop windows. I can do this simultaneously without any problems. The situation changes completely when the surface is covered with ice. I feel that my feet don't have the same grip on the surface as usual. I immediately change my movement pattern. I walk more slowly overall, put my foot down carefully and only lift my other leg when I'm sure I won't slip. In addition, I now look closely at where I put my foot instead of continuing to look at the shop window displays.

The relevance of the tactile-kinesthetic system is described in the context of perceptual organisation (K7); (6)⁠⁠. Affolters consideration of the tactile and the kinesthetic system as one, integrated system can be traced back to Harper (34)⁠⁠. In this context, it is important to emphasise that little is known about the role of this system in comparison to other sensory modalities (2, 7, 33)⁠.

Statement K6 states that theories and findings from related sciences play an important role in the further development of the Affolter Model and that these are integrated wherever possible (33). There was initially disagreement among the participants regarding this statement. Some participants noted that not enough findings from related sciences have been integrated to date. Other participants were in favour of excluding specific theories. Only a modified version of statement K6 achieved consensus. Statement K12 describes the target group that can be identified and treated using Tactual Interaction Therapy.

#### 4.2.2 Principles

At the principles level, statements about value orientations, convictions, design ideas and overarching modes of action in therapeutic practice (statements in Table 3) are presented (Table 3).

The participants consider behavioural observation to be a central principle of intervention according to the Affolter Model. There is agreement that behavioural observations allow conclusions to be drawn about the (developmental) status (P1), the organisation of perception and the possibility of interaction of the person concerned (P2); (6)⁠. Video recordings can be used to analyse behaviour with regard to the organisation of perception and the complexity of a situation (P7). The results of behavioural observation are recorded and presented in various ways (P8); (6, 17)⁠. Conclusions are drawn from behavioural observations. These determine the therapeutic goals and the next steps (P4). Some participants emphasised that although video documentation (P7) and visual representations provide important information, these can only rarely be used outside of courses due to limited time.

The design of the therapy is orientated towards the developmental level of the person concerned (P3) with the aim of stimulating information-seeking and problem-solving processes (P5); (6)⁠. Everyday life activities that are relevant and meaningful for the person are organised (P9); (4, 6). Statement P12 states that when working with familiar and unfamiliar events, it is important to be aware of what might be difficult for the person concerned, to avoid stress. However, this statement did not achieve unanimity consensus. This could be due to the fact that there is not yet sufficient clarity among practitioners about the role of stress during treatment.

Statement P6 expresses the conviction that learning and development take place at the level of understanding and that the level of understanding is more comprehensive than production (execution of activities) (4, 6)⁠⁠. Affolter (7), p. 221f. describes it similarly: »Understanding is a prerequisite for learning. If I do not understand something, I cannot store it. Therefore, I cannot learn.«

Regarding the implementation of therapy, it is stated that this should ideally be carried out on an interprofessional basis (P10) and that relatives should be involved wherever possible (P11).

#### 4.2.3 Methods

Central questions at the level of methods are which actions can be used to achieve therapeutic goals and how therapists and practitioners structurise and organise the treatment (cf. statements in Table 4) (Table 4).

The expert panel considers the non-verbal communication of tactual information in everyday activities to be a central intervention (M1); (35)⁠. Verbalisation only takes place after therapeutic guiding (M3); (4, 6)⁠⁠. An explanation for this approach can be found in Affolter (7)⁠⁠, p. 236: »Solving problems requires tactile-kinesthetic information. Therefore, it is important to pay full attention to the tactile-kinesthetic input during the performance of an event. Talking becomes unimportant; it can even be obstructive. It distracts attention from the tactile-kinesthetic input and disrupts the guiding.« There is consensus that it is possible to cope with everyday activities in an individual or group setting without speaking (M4); (6, 17)⁠⁠. Another method in treatment is to adapt the complexity of guidance to the person concerned in order to stimulate problem-solving processes and the associated hypothesising (M2); (4).

Statement P11 states that relatives should be involved as much as possible. Statement M5 explains how relatives can be specifically involved (4, 6)⁠⁠. In this statement, it was reported back that it is important to provide intensive support for the implementation with relatives so that they feel confident in the application of non-verbal work.

#### 4.2.4 Techniques

The level of techniques includes the description of specific tools for application (cf. statements in Table 5). Designing the environment plays an important role in therapy. The environment should be designed to be stable and as natural as possible for the person concerned. Aids such as positioning materials can be used for this purpose (6)⁠⁠. When selecting the activity, there was a consensus that individual needs must be considered, it must be suitable and relevant for the person (T2); ⁠⁠(6, 36)⁠. The aspect of sensing topological relationships is also taken into account when selecting the activity (T4); (6)⁠⁠. During therapy, »magic«, i.e., the execution of partial steps by the caregiver, is avoided. This means that all topological changes are experienced in collaboration with the person concerned (T7) (Table 5).

A distinction is made between three types of guiding—nursing guiding, elementary guiding and intensive guiding. The choice of guiding type depends on the functional level of the person concerned and the everyday events (T4); (6)⁠. In the case of intensive guiding, some participants reported that they were not familiar with this type of guiding or that it was hardly ever used. This fact was accommodated in a new modified version of the statement.

Regarding guiding, the participants agree that the person concerned can carry out parts of the action independently if the execution is of sufficient quality (T8). Statement T6 contains an explanation of what the technique of guiding involves (6)⁠⁠. Statement T9 also emphasises that legs and feet should also be included in elementary guiding, if this makes sense in the event (T9). The importance of speaking has already been addressed in statements M1 and M3. A concretisation of this can be found in statement T5 (37)⁠⁠.

### 4.3 Limitations and strengths

This study has some limitations. Firstly, the expert panel was limited to selected members of the APW with many years of experience and training in the application of the Affolter Model. This also meant a language limitation to German and French-speaking participants. Nevertheless, it can be assumed that members of the APW were best qualified to participate in the expert panel due to their regular meetings, exchanges and further training. The translations and the co-operation of the international project group showed that differentiated use of language is essential for understanding the statements and thus for reaching consensus among the participants. The interprofessional and international composition of the project group proved to be a great advantage. On the one hand, it made it possible to translate the surveys in a technically correct manner and thus to include the participants of the expert panel with a mother tongue other than German. Due to the different professional backgrounds, it was also possible to account for many profession-specific aspects of different areas of application of the Affolter Model when developing the statements. Statements that dealt with specific aspects such as development, language and motor skills benefited in their formulation from the fact that various professional groups were represented in the project group. A further strength is that only three of the 40 participants dropped out between rounds. This increases the internal validity of the study.⁠

The methodological approach had to be adapted after the first round of the survey. Originally, the project group did not plan for the expert panel to be able to adapt the content and language in the form of new versions of statements. However, due to numerous suggestions for changes to the statements, these were integrated. This adaptation proved to be a strength of this study. It made it possible to exert greater influence and thus possibly increase the motivation of the expert panel. This was demonstrated by the fact that there was a clear preference for the new versions. The challenge with the new versions was that they were not always formulated by the expert group as concrete/precise statements. The project group therefore also tried to integrate incomplete statements or comments as objectively as possible in the form of new versions of the statement.

The five-point Likert scale used, is an ordinal scale level (cf. Realisation). The calculation of statistical parameters from ordinal-scaled variables is controversial (38)⁠⁠. One point of criticism is that ordinal-scaled variables must not be treated like interval-scaled variables (39)⁠. There is a risk that the distances between the individual categories are not interpreted as being of equal size. This risk was addressed by additionally calculating the median. The use of Likert scales also harbours the risk of harsh or mild judgements and a tendency towards the middle (39)⁠. Due to the high level of agreement in this study, it can be assumed that there is a tendency towards a lenient response.

The purpose of providing individualized feedback after each round was to encourage participants to reflect more deeply on the statements and to better prepare for the subsequent round. However, this approach also introduces the risk of an anchoring effect, as exposure to the group's response tendencies could disproportionately influence participants' later ratings. To mitigate this risk, we delivered feedback as controlled, anonymized statistical summaries—such as mean and median—thus reducing the potential influence of dominant positions.

Goodman (40)⁠⁠, Niederberger and Renn (41)⁠ stated that the results of a Delphi process are heavily dependent on the composition of the expert panel, the project group (monitoring team) and ultimately also on the definition of the consensus. In this study, various professional perspectives were included in the description of the core domains by means of an inter-professional project group and expert panel. The representation of the different professional groups was too small to carry out profession-specific data analyses. In future, this issue could be examined in more detail in order to further strengthen the interprofessional aspects of the model.

The authors interpret that, in combination with the high threshold value of 80% and the consideration of the median, it can be assumed that these statements are representative of the Affolter Model.

### 4.4 Clinical implications and research perspectives

This study is the first to offer a contemporary mapping of the Affolter Model and the therein integrated principles, methods and techniques. This description can be used to introduce the Affolter Model to a wider audience in the context of publications, further training, education and courses, thus contributing to its dissemination.⁠

The studies by Affolter et al. (11)⁠, Blak Lund et al. (12)⁠⁠ and Schaub et al. (13)⁠ show which study designs are possible for assessing effectiveness and which approaches can be used to record changes. In the interest of best evidence, further studies with larger samples of people with perceptual disorders of different origins and different age groups are needed in the future. In this regard, creating guidelines for conducting case studies at different locations is feasible. Consequently, it is important to develop a common treatment protocol in which the assumed effective factors and treatment outcomes are defined. This can improve and simplify the replication and synthesis of case studies. On the other hand, the further development and validation of existing measurement instruments for documenting behaviour and the level of function and activity is necessary to compare the effect of therapeutic interventions.

## 5 Conclusion

The use of a modified Delphi process proved to be an effective way of integrating expert knowledge into the description of the Affolter Model. The initial statements underwent a reformulation and restructuring process, and new content could be included in the four survey rounds. The clear preference for the newly created versions illustrates the benefits and necessity of summarising and describing expert knowledge using a Delphi process with multiple survey rounds.

The results of this study should not be seen as conclusive, but rather a continues iterative process of understanding of the Affolter Model. The content of the statements is therefore intended to encourage users to engage in a critical exchange, reflect on the Affolter Model and its further development. In addition, the description should clarify how the Affolter Model differs from other rehabilitative concepts and approaches and what the advantages are. The results of this study provide an overview of the core domains of the Affolter Model. This can be used to analyse and evaluate the clinical effectiveness of the Affolter Model as it is currently understood. This should ensure the quality of the range of Tactual Interaction Therapy for children, adolescents, and adults in the future**.** Last but not least, the description of the content of the Affolter Model provides a basis for practical work and teaching in courses or the instruction of relatives and thus ensures adherence to its application.

## Data Availability

The raw data supporting the conclusions of this article will be made available by the authors, without undue reservation.
