# Supplementary material for: Defining the key elements of the Affolter Model® in a multiprofessional Delphi study: a first step toward evidence-based Tactual Interaction Therapy
Source: Front Rehabil Sci. 2025 Oct 10;6:1624757. doi: 10.3389/fresc.2025.1624757 (PMC12549633; doi:10.3389/fresc.2025.1624757)
Supplement: Supplementary file 1 [file Presentation1.pdf]

Appendix A

Advertisement

Einladung zur Teilnahme am Projekt:

Die Beschreibung des Affolter-Modells® im Rahmen eines Delphi-Verfahrens

1. Beschreibung des Projekts

Das Affolter-Modell® wird in der Neurorehabilitation und in sonder- und heilpädagogischen Arbeitsfeldern eingesetzt und von verschiedenen Berufsgruppen bei Kindern und Erwachsenen mit angeborener oder erworbener gestörter Organisation der Wahrnehmungsleistungen praktiziert. Bei dem Modell handelt es sich um ein Lern- und Entwicklungsmodell und um eine Therapiemethode (www.wahrnehmung.ch, 2021). Das Lern- und Entwicklungsmodell basiert auf der Grundannahme, dass die Entwicklung eines Menschen in Interaktion mit seiner Umwelt stattfindet (Hofer, 2009). Gestützt auf dieses Entwicklungsmodell wurde von Frau Dr. Affolter und Dr. Bischofberger ein Therapiekonzept entwickelt, welche Personen mit einer Wahrnehmungsstörung bei der Informationssuche innerhalb von problembehafteten Alltagsgeschehnissen unterstützt (www.wahrnehmung.ch, 2021). Ziel ist das (Wieder)Erlernen von perzeptiven und kognitiven Fähigkeiten für grösstmögliche Selbständigkeit und Teilhabe im Alltag.

1.1 Hintergrund der Untersuchung: Trotz der breiten Anwendung des Affolter-Modells® im therapeutischen und heilpädagogischen/pädagogischen Alltag, fehlt bisher der wissenschaftliche Nachweis der Wirksamkeit im Sinne der Best-Evidence. Dies ist problematisch im Hinblick darauf, dass insbesondere das Gesundheitspersonal sowie auch der (heil-/sonder) pädagogische Bereich zunehmend aufgefordert wird, die Wirksamkeit ihrer Interventionen und der angewendeten therapeutischen und pädagogischen Konzepte mit wissenschaftlichen Belegen abzusichern. (Christopher & Weise, 2013).

1.2 Ziel dieser Studie: Um zukünftig die Effektivität des Affolter-Modells® in Untersuchungen validieren zu können, ist der Zweck dieser Studie, die Inhalte des Konzepts und die darin integrierten Prinzipien, Methoden, und Techniken innerhalb eines mehrstufigen Konsensusverfahrens zu bestimmen. Damit soll diese Untersuchung eine Grundlage für weitere Forschung sein. Dadurch soll die Qualität des Angebots an Gespürter Interaktionstherapie für Kinder, Jugendliche und Erwachsene mit gestörter Organisation der Wahrnehmungsleistungen in Neurorehabilitations- und Nachsorgeeinrichtungen, Pflege/Wohnheime, (Sonder) Schulen und für ambulante Therapieangebote gesichert werden.

2. Projektteam

Diese Untersuchung wird von APW-anerkannten Instruktor*innen des Affolter-Modells® (Daniela Jakobsen, DK/ Sabine Augstein, CH/ Laurent Munch, CH/ und Frank Roelandt, D) in Zusammenarbeit mit Tamarith Schlunegger (Universität Basel) durchgeführt. Das Projekt ist Gegenstand der Masterarbeit von Tamarith Schlunegger und wird von Prof. Dr. Karin Hediger (Universität Basel) betreut.

Eine weiter am Projekt beteiligte Institution ist die REHAB Klinik Basel, sowie die Arbeitsgemeinschaft pro Wahrnehmung (APW) mit Sitz in der Schweiz.

3. Ihre Teilnahme an der Untersuchung

3.1 Weshalb wir Sie für eine Teilnahme an unserer Untersuchung anfragen: Diese Untersuchung wird mithilfe des Delphi-Verfahrens durchgeführt. Bei diesem handelt es sich um einen strukturierten Gruppenkommunikationsprozess. Innerhalb des Verfahrens können komplexe Sachverhalte, über die unvollständiges und/ oder unsicheres Wissen vorhanden ist, durch Expert*innen schrittweise beurteilt werden. Ein Delphi-Verfahren wird klassischerweise in drei bis vier Runden in anonymisierter Form durchgeführt. Das Ziel ist es einen größtmöglichen Konsens der Expert*innengruppe zu erreichen (Niederberger & Renn, 2019).

Sie werden für diese Untersuchung kontaktiert, da Sie;

1. zu der Anwender*innengruppe der Gespürten Interaktionstherapie/ Affolter Modell® gehören,
2. den Grundkurs im Affolter Modell® absolviert haben,
3. mindestens über 5 Jahre Berufserfahrung in der Anwendung des Affolter-Modells® verfügen und
4. über eine gültige Qualifikation verfügen (mit Ausnahme: ehemalige, pensionierte Instruktor*innen werden ebenfalls angefragt).

3.2 Ablauf der Untersuchung: Um einen grösstmöglichen Konsensus zu erreichen, werden Ihnen im Rahmen der Untersuchung Aussagen (Statements) zum Affolter-Modell® präsentiert. Ihre Aufgabe wird es sein zu diesen auf einer fünfstufigen Skala Ihre Zustimmung beziehungsweise Ablehnung anzugeben. Ausserdem haben Sie die Möglichkeit in den ersten zwei Runden zusätzliche Statements zu generieren. In der untenstehenden Abbildung ist der schematische Ablauf dieser Untersuchung abgebildet.

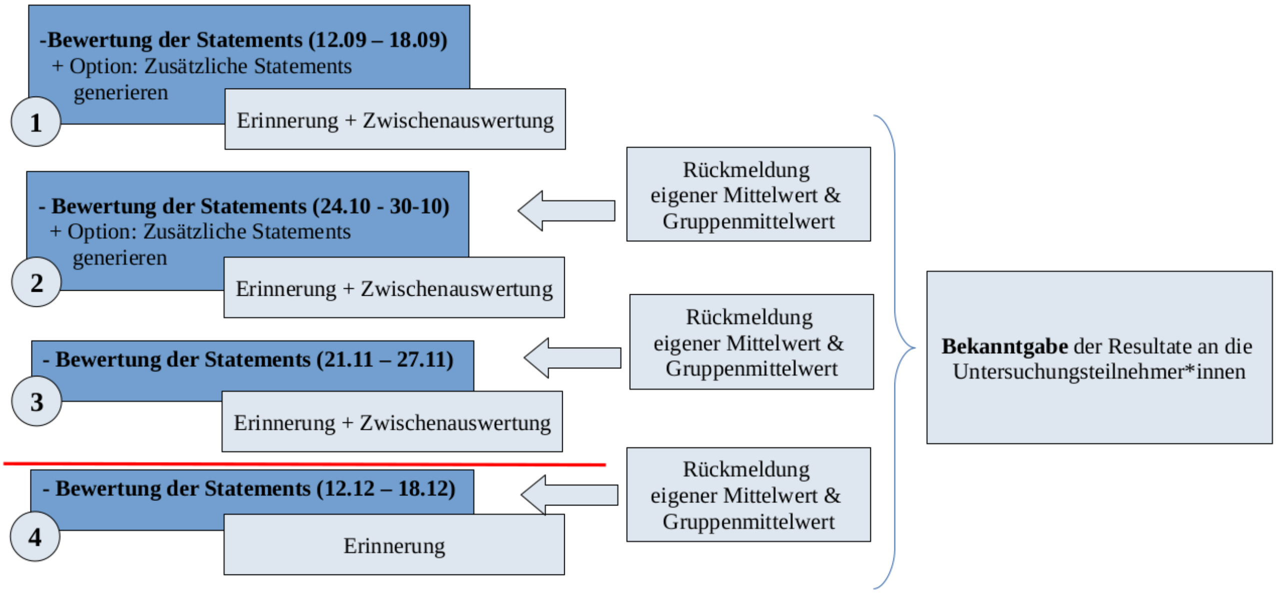

Ab der zweiten Runde erhalten Sie eine Rückmeldung über die Ergebnisse der vorherigen Runde. Somit erhalten Sie ein Feedback über Ihr Urteilverhalten sowie über das der anderen Studienteilnehmer*innen. Anschliessend haben Sie die Möglichkeit, in der nächsten Runde Ihr Urteilverhalten in Anbetracht der vorherigen Runden zu überdenken und gegebenenfalls anzupassen.

3.3 Vorteile einer Teilnahme: Indem Sie Ihr Wissen teilen, leisten Sie einen wichtigen Beitrag zur Definition und Eingrenzung der Affolter-Methode® und ermöglichen damit, einen Grundstein für weitere Forschungsarbeiten zu legen.

Um den zeitlichen Aufwand einzugrenzen, wird die Untersuchung online durchgeführt. Somit können Sie bequem von Zuhause aus teilnehmen. Die Teilnahme an diesem Projekt kann nicht finanziell vergütet werden.

3.4 Risiken der Teilnahme: Es entstehen keine absehbaren Risiken für Sie durch eine Teilnahme an dieser Befragung. Ihre Anonymität gegenüber den anderen Teilnehmer*innen ist gewährleistet. Indem Sie ihre Mail-Adresse angeben, ermöglichen Sie Tamarith Schlunegger, Ihnen ihre Resultate für die nächsten Runden zu übermitteln. Ihre Antworten werden in einer nicht Re-Identifizierbaren Form (als Gruppenmittelwert aller Teilnehmenden) weitergeleitet und möglicherweise für eine wissenschaftliche Publikation verwendet. In beiden Fällen ist es nicht möglich, Rückschlüsse auf Ihre Person zu ziehen.

3. Die nächsten Schritte und Kontaktaufnahme

Die Teilnahme an dieser Untersuchung ist freiwillig. Sie können jederzeit und ohne Angabe von Gründen Ihre Zustimmung zur Teilnahme widerrufen, ohne dass Ihnen deswegen Nachteile entstehen. Für den Erfolg (Datenerhebung und Auswertung) dieser Untersuchung ist jedoch eine vollständige Teilnahme (3-4 Umfragerunden) Ihrerseits optimal. Mit der Teilnahme erklären Sie sich zur Weitergabe Ihrer E-Mail-Adresse an Tamarith Schlunegger bereit. Dies ist notwendig, damit Tamarith Schlunegger Sie für die Umfrage kontaktieren kann. Ihre Adresse wird nur für den Zweck dieser Untersuchung verwendet und nach Ende der Befragung von der Studienleiterin gelöscht.

3.1 Zeitplan: In der nachfolgenden Abbildung ist der Zeitplan der Untersuchung abgebildet. Die Befragung findet ab Mitte September einmal im Monat statt. Um an der Befragung teilzunehmen, erhalten Sie jeweils eine Woche Zeit. Der Umfragelink, welcher eine Teilnahme am Verfahren ermöglicht, wird Ihnen immer am Montagmorgen via Mail zugesendet. Der zeitliche Aufwand für die erste Teilnahme beträgt rund *eineinhalb Stunden*. In den Folgerunden wird erwartet, dass die Bearbeitungszeit für die Umfrage abnimmt. Abhängig von den Zwischenresultaten nach der dritten Runde wird eine vierte Runde durchgeführt.

Runden	Zeitfenster für die Teilnahme
1	12.09.22 – 18.09.22
2	24.10.22 – 30.10.22
3	21.11.22 – 27.11.22
4	12.12.22 – 18.12.22

Wir würden uns sehr über Ihre Teilnahme freuen! Falls Sie sich zu einer Teilnahme entscheiden, kontaktieren Sie bitte:

Tamarith Schlunegger
t.schlunegger@stud.unibas.ch

Bitte geben Sie uns Bescheid, ob Sie über die Resultate der Untersuchung informiert werden möchten.
Bei Fragen oder Unklarheiten zur Untersuchung dürfen Sie jederzeit Kontakt aufnehmen.

Freundliche Grüße

Daniela Jakobsen 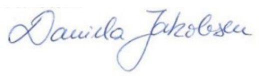

Frank Roelandt 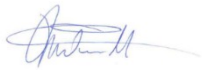

Laurent Munch 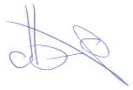

Sabine Augstein 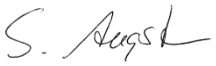

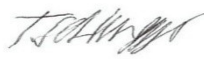

Tamarith Schlunegger

Appendix B

LimeSurvey (KPTI) - 1. Runde: Die Beschreibung des Affolter-Modells® https://survey-1.psychologie.unibas.ch/kpti/index.php/admin/printablesurvey/s...

1. Runde: Die Beschreibung des Affolter-Modells®

Herzlich **Willkommen** zur ersten Runde der **Beschreibung des Affolter- Modells®** im Rahmen des Delphi-Verfahrens.

Mit ihrer Teilnahme unterstützen Sie dieses Forschungsprojekt, welches zum Ziel hat, die Kerndomänen des Affolter-Modells® und dessen Inhalte innerhalb des mehrstufigen Konsensusverfahrens zu beschreiben.

In dieser Umfrage sind 44 Fragen enthalten.

Demographische Angaben

Im ersten Abschnitt der Befragung werden Ihnen Fragen zu Ihrem Beruf sowie Ihrer Person gestellt. Bitte beantworten Sie jede Frage so ehrlich und genau wie Sie können.

Bitte geben Sie Ihr Geschlecht an.

Bitte wählen Sie nur eine der folgenden Antworten aus:

- ○ Weiblich
- ○ männlich

1 von 49 06.03.23, 19:00

LimeSurvey (KPTI) - 1. Runde: Die Beschreibung des Affolter-Modells® https://survey-1.psychologie.unibas.ch/kpti/index.php/admin/printablesurvey/s...

Bitte geben Sie Ihr Alter in Jahren an. *

Bitte geben Sie Ihre Antwort hier ein:

In welchem Land sind (waren) Sie erwerbstätig? *

Bitte wählen Sie eine der folgenden Antworten:
Bitte wählen Sie nur eine der folgenden Antworten aus:

- ○ Schweiz
- ○ Deutschland
- ○ Frankreich
- ○ Belgien
- ○ anderes Land

2 von 49 06.03.23, 19:00

LimeSurvey (KPTI) - 1. Runde: Die Beschreibung des Affolter-Modells® https://survey-1.psychologie.unibas.ch/kpti/index.php/admin/printablesurvey/s...

Welchen der hier aufgelisteten Berufe üben Sie derzeit aus? (Mehrfachnennungen möglich) *

Bitte wählen Sie die zutreffenden Antworten aus:
Bitte wählen Sie alle zutreffenden Antworten aus:

- ☐ Physiotherapeut/in
- ☐ Ergotherapeut/in
- ☐ Logopäd/in
- ☐ Sozialpädagog/in
- ☐ Heilpädagog/in
- ☐ Pflegfachfrau, Pflegefachmann
- ☐ Fachfrau Gesundheit, Fachmann Gesundheit
- ☐ Fachfrau Betreuung, Fachmann Betreuung
- ☐ Psycholog/in
- ☐ anderer Beruf/ andere Tätigkeit:

Bitte wählen Sie die Berufsbezeichnung, welche am besten zu Ihrer aktuellen Tätigkeit passt. Sollte keine der aufgelisteten Tätigkeitsformen auf Ihre Situation zutreffen, können Sie diese unter der Option "anderer Beruf/andere Tätigkeit" ergänzen.

3 von 49 06.03.23, 19:00

LimeSurvey (KPTI) - 1. Runde: Die Beschreibung des Affolter-Modells® https://survey-1.psychologie.unibas.ch/kpti/index.php/admin/printablesurvey/s...

Welche der folgenden Arbeitszeiten beschreibt Ihren momentanen Beschäftigungsgrad am besten?
*

Bitte wählen Sie eine der folgenden Antworten:
Bitte wählen Sie nur eine der folgenden Antworten aus:

- ○ 100%
- ○ 80-100%
- ○ 60-80%
- ○ weniger als 60%
- ○ anderes

4 von 49 06.03.23, 19:00

LimeSurvey (KPTI) - 1. Runde: Die Beschreibung des Affolter-Modells® https://survey-1.psychologie.unibas.ch/kpti/index.php/admin/printablesurvey/s...

In welchem dieser Bereiche ist Ihre aktuelle Anstellung angesiedelt? *

Bitte wählen Sie eine der folgenden Antworten:
Bitte wählen Sie nur eine der folgenden Antworten aus:

- ○ Krankenhaus
- ○ Rehabilitationsklinik
- ○ Heilpädagogische Einrichtung
- ○ anderes

Bitte wählen Sie die Option aus, die auf Ihre Anstellung am zutreffendsten ist. Falls keine Vorgabeantwort passt, können Sie die passende Bezeichnung unter "anderes" konkretisieren.

Über wie viele Jahre Berufserfahrung verfügen Sie in Ihrem Beruf? *

Bitte wählen Sie eine der folgenden Antworten:
Bitte wählen Sie nur eine der folgenden Antworten aus:

- ○ 0-5 Jahre
- ○ 5-10 Jahre
- ○ 10-15 Jahre
- ○ 15-20 Jahre
- ○ mehr als 20 Jahre

5 von 49 06.03.23, 19:00

LimeSurvey (KPTI) - 1. Runde: Die Beschreibung des Affolter-Modells® https://survey-1.psychologie.unibas.ch/kpti/index.php/admin/printablesurvey/s...

Seit wie vielen Jahren arbeiten Sie mit dem Affolter-Modell®? *

Bitte wählen Sie eine der folgenden Antworten:
Bitte wählen Sie nur eine der folgenden Antworten aus:

- ○ 0-5 Jahre
- ○ 5-10 Jahre
- ○ 10-15 Jahre
- ○ 15-20 Jahre
- ○ über 20 Jahre

Beurteilung der Statements

Sie erhalten nun erneut eine Sammlung an Statements (Aussagen) zur Beurteilung Ihrer Zustimmung zur Richtigkeit. Diese Statements konnten im Rahmen der 1. **Runde** von Ihnen erstellt werden. Diese Statements werden Ihnen erneut nach dem Ordnungssystem von Ritter und Welling (2011) präsentiert.

In der nachfolgenden Abbildung sehen Sie das verwendete Ordnungssystem.

Insgesamt enthält dieses vier verschachtelte Ebenen:

- 1. Konzept
- 2. Prinzipien
- 3. Methoden
- 4. Techniken

6 von 49 06.03.23, 19:00

LimeSurvey (KPTI) - 1. Runde: Die Beschreibung des Affolter-Modells® https://survey-1.psychologie.unibas.ch/kpti/index.php/admin/printablesurvey/s...

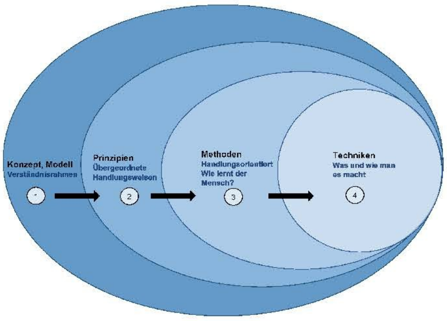

7 von 49 06.03.23, 19:00

LimeSurvey (KPTI) - 1. Runde: Die Beschreibung des Affolter-Modells® https://survey-1.psychologie.unibas.ch/kpti/index.php/admin/printablesurvey/s...

Im nachfolgenden Abschnitt ist Ihre Aufgabe, bei jedem Statement Ihre Zustimmung beziehungsweise Ablehnung anzugeben. Bei jedem Statement haben Sie die Möglichkeit, aus fünf Antwortoptionen auszuwählen.

Diese lauten:

- 0 = Stimme überhaupt nicht zu
- 1 = Stimme wenig zu
- 2 = Stimme mittelmässig zu
- 3 = Stimme ziemlich zu
- 4 = Stimme voll und ganz zu

Die Statements werden Ihnen gruppiert zur jeweiligen Ebene präsentiert. Die Statements, die zu diesem Zeitpunkt vorhanden sind, erheben nicht den Anspruch an Vollständigkeit. Deshalb haben Sie am Ende jeder Umfrageseite zusätzlich die Möglichkeit, selbst Statements zu formulieren. Damit soll sichergestellt werden, dass mithilfe dieser Untersuchung möglichst alle Aspekte des Affolter-Modells® repräsentiert werden.

1. Beurteilungsebene: Konzept

Herzlich Willkommen zum ersten Abschnitt zur Beurteilung der Statements. In diesem werden Ihnen die Statements präsentiert, die der Konzeptebene zugeordnet werden.

Charakteristisch für die Ebene "Konzept" sind die Fragen;

- Was sind die zentralen Begriffe und Grundaussagen des Affolter-Modells®?
- Welche Ausgangsideen sind Grundlage des Affolter-Modells®?

8 von 49 06.03.23, 19:00

Sample questionnaire (first round)

- Auf welche wissenschaftlichen Modelle oder (Alltags-)theorien wird im Affolter-Modell® zurückgegriffen

Achtung: Zum besseren Verständnis ist in dieser Abbildung eine gekürzte Form der Statements abgebildet. Ihre Aufgabe ist zu beurteilen, inwiefern Sie diesen Aussagen zustimmen beziehungsweise ablehnen und bei Bedarf weitere Statements zu formulieren.

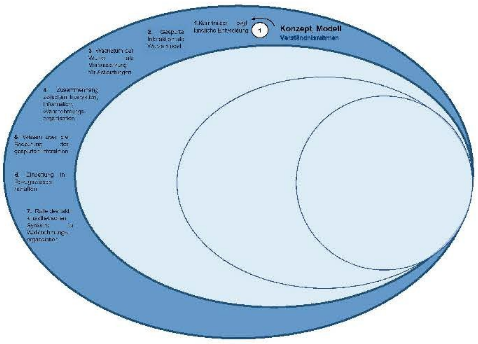

Wir bitten Sie, alle Statements nach der Frage zu beurteilen, inwiefern Sie diese als Bestandteil des Affolter-Modells® verstehen. Für die Beurteilung gibt es keine **richtige oder falsche** Antwort. Wir sind an Ihrer persönlichen Meinung und Einschätzung interessiert. Deshalb bitten wir Sie auch, sich nicht mit Ihren Kolleg*innen über inhaltliche Aspekte dieser Umfrage auszutauschen.

1.1 Erkenntnisse über die kindliche Entwicklung bzgl. der gespürten Interaktion im Alltag, der Wahrnehmungsorganisation, der Bewegung, der Sprache, der Kommunikation und der sozialen Interaktion sind wichtige Grundlagen im Affolter Modell®.
*

❶ Bitte wählen Sie eine der folgenden Antworten:
Bitte wählen Sie nur eine der folgenden Antworten aus:

- ◯ Stimme überhaupt nicht zu
- ◯ Stimme wenig zu
- ◯ Stimme mittelmässig zu
- ◯ Stimme ziemlich zu
- ◯ Stimme voll und ganz zu

1.2 Die gespürte Interaktion in Alltagsgeschehnissen stellt die Wurzel der Entwicklung des Menschen dar (Wurzelmodell).

*

❶ Bitte wählen Sie eine der folgenden Antworten:
Bitte wählen Sie nur eine der folgenden Antworten aus:

- ◯ Stimme überhaupt nicht zu
- ◯ Stimme wenig zu
- ◯ Stimme mittelmässig zu
- ◯ Stimme ziemlich zu
- ◯ Stimme voll und ganz zu

1.3 Ein Repertoire gespürter Interaktionserfahrungen im Alltag führt zum Wachstum der Wurzel als Voraussetzung für das Entstehen von Astleistungen, z. B. sozialer Leistungen.
*

❶ Bitte wählen Sie eine der folgenden Antworten:
Bitte wählen Sie nur eine der folgenden Antworten aus:

- ◯ Stimme überhaupt nicht zu
- ◯ Stimme wenig zu
- ◯ Stimme mittelmässig zu
- ◯ Stimme ziemlich zu
- ◯ Stimme voll und ganz zu

1.4 Es bestehen Zusammenhänge zwischen Interaktion, Information und der damit verbundenen Wahrnehmungsorganisation. Diese sind unerlässliche Grundlagen im Affolter Modell®.
*

❶ Bitte wählen Sie eine der folgenden Antworten:
Bitte wählen Sie nur eine der folgenden Antworten aus:

- ◯ Stimme überhaupt nicht zu
- ◯ Stimme wenig zu
- ◯ Stimme mittelmässig zu
- ◯ Stimme ziemlich zu
- ◯ Stimme voll und ganz zu

1.5 Das Wissen aus der Wahrnehmungspsychologie, insbesondere über die Rolle der gespürten Interaktion innerhalb der intermodalen Wahrnehmungsorganisation sind unerlässliche Grundlagen im Affolter Modell®.
*

❶ Bitte wählen Sie eine der folgenden Antworten:
Bitte wählen Sie nur eine der folgenden Antworten aus:

- ◯ Stimme überhaupt nicht zu
- ◯ Stimme wenig zu
- ◯ Stimme mittelmässig zu
- ◯ Stimme ziemlich zu
- ◯ Stimme voll und ganz zu

1.6 Theorien verschiedener Bezugswissenschaften und relevante Erkenntnisse (Neurorehabilitation, ICF, Embodiment, Neuroplastizität, (Sprach Entwicklungspsychologie, Lernpsychologie) werden in der Weiterentwicklung des Affolter Modells® integriert.
*

❶ Bitte wählen Sie eine der folgenden Antworten:
Bitte wählen Sie nur eine der folgenden Antworten aus:

- ◯ Stimme überhaupt nicht zu
- ◯ Stimme wenig zu
- ◯ Stimme mittelmässig zu
- ◯ Stimme ziemlich zu
- ◯ Stimme voll und ganz zu

1.7 Dem taktil-kinästhetischen System wird im Affolter-Modell® eine besondere Bedeutung bei der Wahrnehmungsorganisation beigemessen. Es ist das einzige Sinnessystem, dass der Mensch gleichzeitig zur Informationssuche und zum Wirken in der Umwelt benutzt.
*

❶ Bitte wählen Sie eine der folgenden Antworten:
Bitte wählen Sie nur eine der folgenden Antworten aus:

- ○ Stimme überhaupt nicht zu
- ○ Stimme wenig zu
- ○ Stimme mittelmässig zu
- ○ Stimme ziemlich zu
- ○ Stimme voll und ganz zu

In diesem Textfeld haben Sie die Möglichkeit, zusätzliche Statements zu formulieren, die Ihrer Meinung nach zur Ebene "Konzept" dazugehören.

Bitte geben Sie Ihre Antwort hier ein:

Falls Sie sich nicht sicher sind, ob das Statement zur Konzeptebene passt, dürfen Sie es selbstverständlich trotzdem an dieser Stelle notieren.

2. Beurteilungsebene: Prinzipien

Im zweiten Schritt bitten wir Sie, Ihre Zustimmung oder Ablehnung zu den Statements der Ebene Prinzipien anzugeben. Charakteristisch für die Ebene "Prinzipien" sind die Fragen;

- Welche Wertorientierungen, Überzeugungen und Gestaltungsideen bestimmen die therapeutische Praxis?
- Welche übergeordneten Handlungsweisen liegen vor?

Für einen ersten Überblick ist in nachstehender Abbildung erneut eine gekürzte Version der Statements abgebildet, die im nächsten Schritt beurteilt werden.

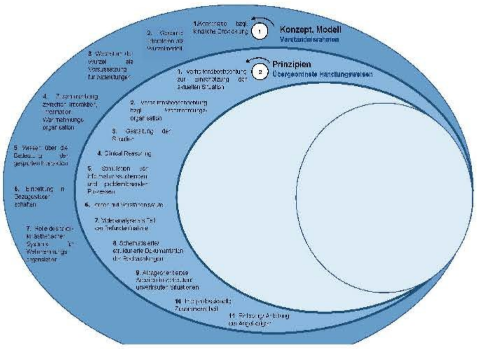

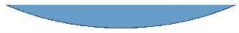

2.1 Um den (Entwicklungs)Stand einer betroffenen Person einzuschätzen, werden Verhaltensbeobachtungen in verschiedenen Situationen gemacht (spontanes Verhalten, Verhalten beim Lösen von Problemen in Geschehnissen des Alltags).
*

❶ Bitte wählen Sie eine der folgenden Antworten:
Bitte wählen Sie nur eine der folgenden Antworten aus:

- ○ Stimme überhaupt nicht zu
- ○ Stimme wenig zu
- ○ Stimme mittelmässig zu
- ○ Stimme ziemlich zu
- ○ Stimme voll und ganz zu

2.2 Verhaltensbeobachtungen (vgl. Statement **2.1**) dienen der Interpretation in Bezug auf die Wahrnehmungsorganisation und der Interaktion der betroffenen Person. Ausserdem werden diese Beobachtungen zur Interpretation des Verständnisses für Alltagsgeschehnisse und der Fähigkeit zur Lösung von Problemen in Alltagsgeschehnissen herangezogen.
*

❶ Bitte wählen Sie eine der folgenden Antworten:
Bitte wählen Sie nur eine der folgenden Antworten aus:

- ○ Stimme überhaupt nicht zu
- ○ Stimme wenig zu
- ○ Stimme mittelmässig zu
- ○ Stimme ziemlich zu
- ○ Stimme voll und ganz zu

2.3 Die Einschätzung des (Entwicklungs-)Standes ist Grundlage für die Auswahl und Gestaltung der Situation. Dies beinhaltet die Auswahl eines adäquaten Alltagsgeschehnisses, die Art des Führens, die Gestaltung der Umwelt und die Wahl der geeigneten Position(en) der Betroffenen.
*

❶ Bitte wählen Sie eine der folgenden Antworten:
Bitte wählen Sie nur eine der folgenden Antworten aus:

- ○ Stimme überhaupt nicht zu
- ○ Stimme wenig zu
- ○ Stimme mittelmässig zu
- ○ Stimme ziemlich zu
- ○ Stimme voll und ganz zu

2.4 Clinical Reasoning: Die Analyse des Verhaltens in verschiedenen Situationen führt zu einer Schlussfolgerung bzgl. der Hauptproblematik der betroffenen Person. Dies ist Voraussetzung für das Formulieren der Therapieziele und Festlegen des konkreten Vorgehens. Das Erreichen dieser Ziele wird kontinuierlich evaluiert und das weitere Vorgehen angepasst*1.

*1: Clinical Reasoning bezeichnet Denk- und Entscheidungsprozesse im Rahmen eines therapeutischen Settings.
*

❶ Bitte wählen Sie eine der folgenden Antworten:
Bitte wählen Sie nur eine der folgenden Antworten aus:

- ○ Stimme überhaupt nicht zu
- ○ Stimme wenig zu
- ○ Stimme mittelmässig zu
- ○ Stimme ziemlich zu
- ○ Stimme voll und ganz zu

2.5 Die Therapie wird so gestaltet, dass dadurch informationssuchende und problemlösende Prozesse angeregt werden.
*

❶ Bitte wählen Sie eine der folgenden Antworten:
Bitte wählen Sie nur eine der folgenden Antworten aus:

- ○ Stimme überhaupt nicht zu
- ○ Stimme wenig zu
- ○ Stimme mittelmässig zu
- ○ Stimme ziemlich zu
- ○ Stimme voll und ganz zu

2.6 Mit den Betroffenen wird auf der Ebene des Verständnisses gearbeitet. Entwicklungs/Lernprozesse finden zuerst auf der Stufe des Verständnisses statt. Es wird davon ausgegangen, dass das Verständnis umfassender als die Stufe der Produktion ist. Auf dem Weg vom Verständnis zur Produktion gibt es noch zwei weitere Stufen: Wiedererkennen und Erwartung.
*

❶ Bitte wählen Sie eine der folgenden Antworten:
Bitte wählen Sie nur eine der folgenden Antworten aus:

- ◯ Stimme überhaupt nicht zu
- ◯ Stimme wenig zu
- ◯ Stimme mittelmässig zu
- ◯ Stimme ziemlich zu
- ◯ Stimme voll und ganz zu

2.7 Videoaufnahmen der Befundaufnahme/Diagnostik sowie von Therapieeinheiten werden für eine genauere Analyse der festgehaltenen Beobachtungen erstellt. In erster Linie interessieren Beobachtungen des Verhaltens, welche bzgl. der Wahrnehmungsorganisation und der Komplexität der Interaktion herangezogen und interpretiert werden.
*

❶ Bitte wählen Sie eine der folgenden Antworten:
Bitte wählen Sie nur eine der folgenden Antworten aus:

- ◯ Stimme überhaupt nicht zu
- ◯ Stimme wenig zu
- ◯ Stimme mittelmässig zu
- ◯ Stimme ziemlich zu
- ◯ Stimme voll und ganz zu

2.8 Beobachtungen aus Videoaufnahmen der Befundaufnahme/Diagnostik sowie von Therapieeinheiten in unterschiedlichen Situationen werden in Form von Skripten, Fenstern, Flussdiagrammen und Strukturbildern dargestellt und zusammengefasst.
*

❶ Bitte wählen Sie eine der folgenden Antworten:
Bitte wählen Sie nur eine der folgenden Antworten aus:

- ◯ Stimme überhaupt nicht zu
- ◯ Stimme wenig zu
- ◯ Stimme mittelmässig zu
- ◯ Stimme ziemlich zu
- ◯ Stimme voll und ganz zu

2.9 Mit der betroffenen Person wird in vertrauten oder unvertrauten Situationen gearbeitet. Diese beinhalten relevante und bedeutungsvolle, vertraute und unvertraute Alltagsgeschehnisse, die den Bedürfnissen und dem aktuellen Stand der betroffenen Person entsprechen.
*

❶ Bitte wählen Sie eine der folgenden Antworten:
Bitte wählen Sie nur eine der folgenden Antworten aus:

- ◯ Stimme überhaupt nicht zu
- ◯ Stimme wenig zu
- ◯ Stimme mittelmässig zu
- ◯ Stimme ziemlich zu
- ◯ Stimme voll und ganz zu

2.10 Im Affolter-Modell® wird idealerweise interprofessionell mit der betroffenen Person gearbeitet. Die Anwender*innen bringen sich hier mit ihren jeweiligen Kernkompetenzen ein, arbeiten zusammen und lernen voneinander, miteinander und übereinander.
*

❶ Bitte wählen Sie eine der folgenden Antworten:
Bitte wählen Sie nur eine der folgenden Antworten aus:

- ◯ Stimme überhaupt nicht zu
- ◯ Stimme wenig zu
- ◯ Stimme mittelmässig zu
- ◯ Stimme ziemlich zu
- ◯ Stimme voll und ganz zu

2.11 Im Affolter-Modell® werden die Angehörigen / Helfer*innen/ Zugehörigen soweit als möglich und relevant einbezogen und angeleitet.

*

❶ Bitte wählen Sie eine der folgenden Antworten:
Bitte wählen Sie nur eine der folgenden Antworten aus:

- ◯ Stimme überhaupt nicht zu
- ◯ Stimme wenig zu
- ◯ Stimme mittelmässig zu
- ◯ Stimme ziemlich zu
- ◯ Stimme voll und ganz zu

In diesem Textfeld haben Sie die Möglichkeit, zusätzliche Statements zu formulieren, die Ihrer Meinung nach zur Ebene **"Prinzipien"** dazugehören.

Bitte geben Sie Ihre Antwort hier ein:

Falls Sie sich nicht sicher sind, ob das Statement zur Ebene **"Prinzipien"** passt, dürfen Sie es selbstverständlich trotzdem an dieser Stelle notieren.

3. Beurteilungsebene: Methoden

Die Statements, die zu der Ebene **"Methoden"** gehören sind handlungsorientiert. Charakteristisch für diese Ebene sind die folgenden Fragen:

- Welche speziellen Verfahrensweisen strukturieren die therapeutischen Abläufe?
- Mit welchen spezifischen therapeutischen Handlungen können die therapeutischen Ziele erreicht werden?

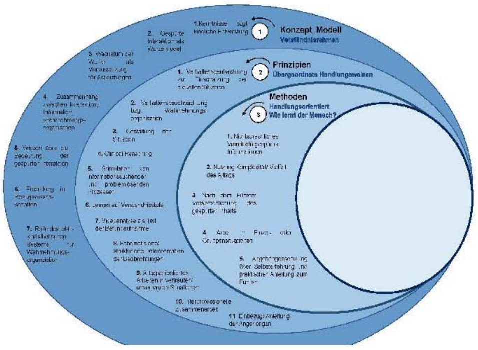

Wir bitten Sie erneut, Ihre Zustimmung bzw. Ablehnung zu den nachfolgenden Statements anzugeben.

3.1 Alltagsgeschehnisse werden mit der betroffenen Person nichtsprachlich über das Vermitteln gespürter Information zur Position und zum Geschehnis durchgeführt. Auftretende Probleme werden nach Möglichkeit mit der betroffenen Person gelöst. Dies geschieht auf der Verständnisstufe.
*

❶ Bitte wählen Sie eine der folgenden Antworten:
Bitte wählen Sie nur eine der folgenden Antworten aus:

- ◯ Stimme überhaupt nicht zu
- ◯ Stimme wenig zu
- ◯ Stimme mittelmässig zu
- ◯ Stimme ziemlich zu
- ◯ Stimme voll und ganz zu

3.2 Die Vielfalt und die unterschiedliche Komplexität von Alltagsgeschehnissen werden genutzt, um das Repertoire der betroffenen Person bei der Problemlösung zu erweitern und problemlösende Prozesse und Hypothesenbildung anzuregen.
*

❶ Bitte wählen Sie eine der folgenden Antworten:
Bitte wählen Sie nur eine der folgenden Antworten aus:

- ◯ Stimme überhaupt nicht zu
- ◯ Stimme wenig zu
- ◯ Stimme mittelmässig zu
- ◯ Stimme ziemlich zu
- ◯ Stimme voll und ganz zu

3.3 Gespürte Alltagsgeschehnisse bzw. Teile davon werden nach dem Führen versprachlicht. Der betroffenen Person werden Formen zum gespürten Inhalt angeboten. Dadurch kann sie auf die gespeicherten Erfahrungen (Inhalt) zurückgreifen und diese mit den angebotenen Formen verknüpfen.
*

❶ Bitte wählen Sie eine der folgenden Antworten:
Bitte wählen Sie nur eine der folgenden Antworten aus:

- ◯ Stimme überhaupt nicht zu
- ◯ Stimme wenig zu
- ◯ Stimme mittelmässig zu
- ◯ Stimme ziemlich zu
- ◯ Stimme voll und ganz zu

3.4 Das nichtsprachliche Bewältigen von Alltagsgeschehnissen kann in Einzel- oder Gruppensituationen stattfinden, abhängig vom aktuellen Stand/ Bedürfnis der betroffenen Person.
*

❶ Bitte wählen Sie eine der folgenden Antworten:
Bitte wählen Sie nur eine der folgenden Antworten aus:

- ◯ Stimme überhaupt nicht zu
- ◯ Stimme wenig zu
- ◯ Stimme mittelmässig zu
- ◯ Stimme ziemlich zu
- ◯ Stimme voll und ganz zu

3.5 Die Angehörigen werden über Teilnahme an der Therapie, Selbsterfahrungen und praktischer Anleitung einbezogen. Die Angehörigenbegleitung kann Führen, die Gestaltung der Umwelt und der Situation sowie das gemeinsame Interpretieren des Verhaltens des Betroffenen beinhalten.
*

❶ Bitte wählen Sie eine der folgenden Antworten:
Bitte wählen Sie nur eine der folgenden Antworten aus:

- ◯ Stimme überhaupt nicht zu
- ◯ Stimme wenig zu
- ◯ Stimme mittelmässig zu
- ◯ Stimme ziemlich zu
- ◯ Stimme voll und ganz zu

In diesem Textfeld haben Sie die Möglichkeit, zusätzliche Statements zu formulieren, die Ihrer Meinung nach zur Ebene **"Methoden"** dazugehören.

Bitte geben Sie Ihre Antwort hier ein:

Falls Sie sich nicht sicher sind, ob das Statement zur Ebene der Methoden passt, dürfen Sie es selbstverständlich trotzdem an dieser Stelle notieren.

4. Beurteilungsebene: Techniken

Nun sind Sie bereits bei der letzten Ebene dieses Fragebogens angelangt.

Für die Ebene **"Techniken"** steht folgende Frage im Vordergrund:

- Welche spezifischen «Werkzeuge» finden Anwendung UND sind eingebettet in ein methodisch strukturiertes Vorgehen?

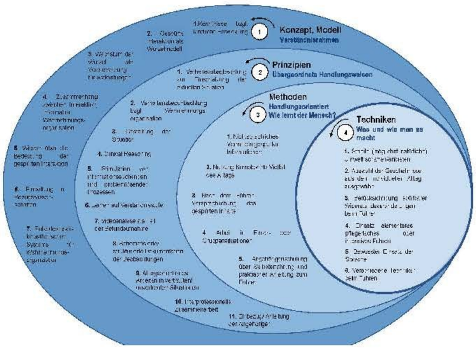

4.1 In der Therapie (und auch sonst im Alltag) wird der betroffenen Person eine möglichst natürliche und stabile Umwelt angeboten. Dies kann auch bedeuten, dass die Umwelt speziell "eingerichtet" wird, dies abhängig vom aktuellen Zustand der betroffenen Person (z.B. Schaffen von Nischen). Je nach Situation kommen Hilfsmittel zum Einsatz (z.B. stabiles Lagerungsmaterial).
*

❶ Bitte wählen Sie eine der folgenden Antworten:
Bitte wählen Sie nur eine der folgenden Antworten aus:

- ◯ Stimme überhaupt nicht zu
- ◯ Stimme wenig zu
- ◯ Stimme mittelmässig zu
- ◯ Stimme ziemlich zu
- ◯ Stimme voll und ganz zu

Herzlichen Dank für die Teilnahme an der ersten Befragungsrunde!

Am 24.10.22 werden Sie den nächsten Link zur zweiten Befragungsrunde erhalten. Bis dahin wünschen wir Ihnen eine gute Zeit.

Bei Fragen melden Sie sich bitte bei: tamarith.schlunegger@stud.unibas.ch

29.11.2022 – 15:56

Übermittlung Ihres ausgefüllten Fragebogens:
Vielen Dank für die Beantwortung des Fragebogens.

Appendix C

Table C1

Strength of agreement with the statements and revisions

	M	*M* [%]	*Mdn*	*SD*	Round	Version	Agreement [%]
K1	3.825	96	4.000	0.501	[1,1]		
K2	3.850	96	4.000	0.362	[1,1]		
K3	3.775	94	4.000	0.530	[1,1]		
K4	3.850	96	4.000	0.362	[1,1]		
K5	3.600	90	4.000	0.672	[1,2]	**5a**	65
K6	3.300	83	3.000	0.687	[1,3]	**6a**	84
K7	3.800	90	4.000	0.464	[1,1]		
K8	3.711	93	4.000	0.515	[2,2]		
K9[a]	2.947	74	3.000	1.207	[2,3]	**9a;**9b;9c	42
K10	3.342	84	4.000	1.192	[2,3]	**10a**;10b	68
K11	3.421	86	4.000	0.889	[3,4]	**11a**;11b	86
K12	3.395	85	4.000	0.887	[2,3]	**12a**	97
P1	3.725	93	4.000	0.716	[1,3]	1a;**1b**	79
P2	3.825	96	4.000	0.446	[1,1]		
P3	3.800	95	4.000	0.516	[1,1]		
P4	3.625	91	4.000	0.705	[1,1]		
P5	3.975	99	4.000	0.158	[1,1]		
P6	3.725	93	4.000	0.554	[1,1]		
P7	3.650	91	4.000	0.580	[1,2]	**7a**	76
P8	3.150	79	3.000	0.864	[1,3]	8a;**8b**	68
P9	3.800	95	4.000	0.464	[2,3]	9a;**9b**	61
P10	3.600	90	4.000	0.591	[1,1]		
P11	3.775	94	4.000	0.530	[1,1]		
P12[a]	2.500	63	3.000	1.180	[2,3]	12a;**12b**	47
M1	3.900	98	4.000	0.304	[1,2]	**1a**	76
M2	3.925	98	4.000	0.350	[1,3]	**2a**	71
M3	3.650	91	4.000	0.580	[1,2]	**3a**	92

M4	3.850	96	4.000	0.427	[1,1]		
M5	3.650	91	4.000	0.736	[1,2]	**5a**	84
T1	3.775	94	4.000	0.530	[1,1]		
T2	3.850	96	4.000	0.362	[1,1]		
T3	3.950	99	4.000	0.221	[1,2]	**3a**	89
T4	3.725	93	4.000	0.554	[1,3]	4a;**4b**	60
T5	3.600	90	4.000	0.632	[1,1]		
T6	3.850	96	4.000	0.427	[1,2]	**6a**	84
T7	2.711	68	3.000	1.228	[2,3]	**7a**;7b;7c	71
T8	3.211	80	3.500	1.044	[3,4]	**8a**	81
T9	3.658	91	4.000	0.847	[2,4]	9a;**9b**	73

Notes. The "Rounds" column shows in which survey round the statement was voted on for the first time and in which round it was last voted on. The "Version" column shows how many different new versions of a statement were submitted for voting. In the case of statement K10, for example, two new versions were created (K10a, K10b). Version 10a, which is highlighted, was favoured by 68% of participants (see column "Agreement [%]").

[a] Statements K9 and P12 did not reach a consensus.
